# Physical Conditions That Determine Choice of Filling-Materials

**Published:** 1898-08

**Authors:** S. Blair Luckie

**Affiliations:** Chester, Pa.


### PHYSICAL CONDITIONS THAT DETERMINE CHOICE OF FILLING-MATERIALS.[1]

BY S. BLAIR LUCKIE, D.D.S., CHESTER, PA.

DIFFERENT materials are in use for repairing the loss of tooth-structure and arresting the progress of dental caries. That no one of them possesses all the necessary properties of a perfect filling is evident, as frequently all are seen in the same mouth, and within the last few years two will be employed in the same cavity with supposed advantage. There must therefore be something to determine the use of one material in preference to the others.

We are all familiar with the expressions "that teeth are too soft or too poorly calcified to be filled with metals, particularly gold; that children's teeth should be filled with plastics until they become sufficient in strength and density to bear filling with some more indestructible material; and the much emphasized remark of the new-departure tripod, in proportion as teeth need filling gold is the worst material to use." "Chalky teeth," "frail teeth," and "soft teeth," and the terms used to designate an opposite condition, such as "hard teeth," "dense teeth," and "strong teeth," seem to express the conditions found at different periods of life better than otherwise could be done, as by long use they have be-

---

[1] Read before the Academy of Stomatology, February 22, 1898.

come familiar exponents of a knowledge obtained from clinical observation.

Assuming that physical conditions should determine the choice of filling material, it may rightly be said that the selection of the proper material with which to fill the cavity constitutes the keystone of operative dentistry, for one who masters the art of determining the condition that indicates what to use is not only skilled in the treatment of dental caries, but has acquired a knowledge of the principle of operative practice.

The conditions that determine the choice of material with which to fill a tooth are general and local. The general conditions, such as physical weakness and an inability to control one's self, or be influenced by the efforts of the operator in that direction, are as important in the consideration as are those conditions found within the tooth itself. The divisions of general conditions are approximately related, one may be dependent upon the other, but patients in either condition may be nervously exhausted by dental operations of a protracted character. Much may depend upon the skill and the ability of the operator to manage such patients, but it must be remembered that the liability of individuals to nervous exhaustion, shock, or collapse from physical or psychical impressions, is not governed by any known law, the consensus of opinions of those surgeons who have studied its causes being that persons are most liable to suffer from it whose nervous system is already in what, for a more appropriate name, is called a state of depression, or if mental perturbation, as fright, precedes an injury or operation, the effect may be the same, though health had been enjoyed up to the time. So collapse or shock is considered of two kinds, mental and corporal. In the first the person may be, to use a common phrase, more frightened than hurt, yet the results may be as serious as though produced by physical injuries. Instances are on record where very slight or no physical injury was received, yet fatal results followed. So mental impressions are considered as potent to give rise to prostration varying in severity as that of traumatic origin. The method of inflicting the injury is important in determining the degree of shock, but fear or terror has as great an etiological importance in its production. In railroad accident, for instance, where no physical injury was received the fear subsequent to the accident has so disturbed the equilibrium of the cerebro-spinal centres as to produce a degree of shock of more than ordinary severity and duration.

As shock has occurred after prolonged dental operations, it is essential that the dentist should consider the tax upon the nervous system of his patient, and for their welfare refrain from operations the protractions of which, through conditions either permanently established in the make-up of the individual or caused by temporary impressions, might produce this undesirable condition, with all its varying discomforts and consequences. Sufficient warning has been given the profession by Dr. Black in the "American System of Dentistry," and by Dr. James Truman in a paper read before the Odontological Society of Pennsylvania a few years ago upon this subject, each dwelling particularly on the condition of prostration following prolonged dental operations. I have, however, been sufficiently impressed with the importance of considering the liability of shock to those patients who have a dread that cannot easily be modified, and who, we are certain, are more frightened than hurt. While their teeth may be in a good condition to receive gold fillings, and be the better preserved thereby, unless fears can be allayed, such permanent work should be substituted by plastics, to be renewed when deficient, or more permanently filled if confidence is gained.

Of the local conditions that determine the selection of filling material may be mentioned the size of cavity, location of cavity, and the organic matter within the tooth. All things being equal, location and size of cavity receive treatment according to manipulative ability and skill; inaccessibility may be readily overcome by one who studies methods to remove obstacles, and thus, to him, fewer interferences occur to hinder than one who recognizes the way to pass obstacles is to use a material of easy introduction and of rapid manipulation. Of local conditions, the organic matter in the tooth is the most important to consider, whether it be that of the pulp or the dentine. If the pulp is encroached upon to such an extent that its life is in danger, plasticity, with protection to the organ, and antiseptics are the requirements, that vital force may have the fullest opportunity to assert itself to make repair. The organic matrix is probably the part that is attracting the most interest and should bring out liberal discussion, for by it are produced the varying conditions observed in the dentine at different periods of life, and which find expression in the terms "hard," "soft," and "poorly calcified" teeth, and also because these conditions seem to have been assailed. Vital properties possessed by the dentine have modified and at times arrested the invasion of caries; and cavities treated by plastic filling-material have produced a change in the resistive power of the whole tooth.

Dr. Black astonished the profession a few years ago with the remark which occurs in his summary of conclusions resulting from a series of experiments concerning the physical character of the human teeth: "There is no basis for the supposition that some teeth are too soft or too poorly calcified to bear filling with gold, or other metal in use for that purpose, since all are found to be abundantly strong," and on page 414 of the *Dental Cosmos*, in which the article appears, he says, "In comparison with the best of granite, dentine is more than one-third stronger," and on page 402, "The greatest good that I could expect to come from this laborious investigation (except as it may affect future studies of caries of the teeth) would be the general abandonment of the idea that the teeth of any patient are so lacking in density as to interfere with reasonable filling operations, with any of the materials now in use. . . . It must, of course, be understood that there may be, and often are, reasons for not immediately using metals in this or that case. The teeth may be hypersensitive, a child may be too young to bear such operations, but the reason for deferring them is not to be found in the softness of the teeth."

To many this seems to be an assertion antagonistic to generally accepted opinions. Yet Dr. Black has presented his conclusions and given the extent as well as the thoroughness of his investigations in a manner that for a long time hardly more than a murmur of non-acceptance could be observed. That Dr. Black has placed himself in a position of safety by allowing a loop of escape is evidenced by the modification that accompanies his expressions. In the last quotation this can be observed in the words, "It must, of course, be understood that there may be, and often are, reasons for not immediately using metals in this or that case. The teeth may be hypersensitive, a child may be too young to bear such operations." It is here, to my mind, he brings us to a point where clinical experience and the results of his investigations may be made to agree, although he does not define his agreement.

The clinician, when he expresses his opinion of the physical state in the terms "too soft" or "too poorly calcified," receives this impression from the condition of the vital portion of the dentine, and Dr. Black, when he qualifies his remarks by the use of the words, "It must, of course, be understood that there may be, and often are, reasons for not immediately using metals in this or that case," recognizes vital conditions and their influences upon the teeth for strength and resistibility to caries, for on page 392 he also says, "After the work I have done with the teeth, I have come to regard the organic matrix of the tooth as much more important than I had formerly considered it. The conditions of the organic matter seem to have much to do with the strength of the tooth, and is a matter that needs further investigation."

Dr. Black's tests show that the teeth of the young are the strongest, as nearly all of the high crushing stresses were found in teeth of young people, and this strength not dependent either on the density or the percentage of lime salts, as density and percentage of lime salts was found to increase with age. From which he deducts the conclusion that the strength seems to depend upon the condition of the organic matrix. It has for years been the practice to treat the teeth of children with plastics until there apparently resulted a changed condition of the dentine, and although in conclusions twelve, thirteen, and fourteen we are to accept "all teeth as abundantly strong to be filled with gold or other metals in use for the purpose," and "what basis there may be for the selection of filling-material is not in the tooth being too frail or too poorly calcified," and, with such knowledge as the investigation has given us "the only basis for the selection of filling-material to classes of cases is the individual operator's judgment as to which he can so manipulate as to make the most perfect filling, considering circumstances, his own skill, and the durability of material." This would indicate to the inexperienced practitioner that with the ability of young patients to endure the tax of a gold filling, it would be just as well to insert a good non-leaking, well-anchored gold filling, and rest assured the best service was given the patient. Compare this with the plastic treatment and it is reasonable, by the knowledge of experience, to conclude that the latter treatment would best conserve the purpose of tooth-filling. By experience as well as by Dr. Black's investigations the conclusion is that the organic matrix of the dentine is of great importance.

It requires but a few years' experience in the practice of operative dentistry to discover that fillings in teeth produce a change in the dentine surrounding them. Where plastics are used we expect to find the dentine, when the proper time arrives for a renewal of the filling, to indicate by the sound of and the sensation transmitted by the cutting instrument, when its edge is passed over the surface, that the conditions are such that material requiring more energy and force to introduce can now be used if other considerations are favorable. On the other side, when teeth of the young with cavity filled with gold, that require force to adapt to the walls, are seen with darkened dentine around the filling, indicating physical injury to the living matter, varying from a short distance from the filling to the disturbance of the function of the pulp. And upon the removal of the filling instrumentation designates a less favorable surface of dentine against which to adapt gold.

Facts that are accepted and demonstrated by scientific investigations with instruments of precision have been announced, perhaps in a suggestive way or what might be plausible to consider, before it was possible to make the instrument with which to demonstrate the facts. Dr. Maynard announced the existence of the dental fibrils before they were discovered by the aid of the microscope. His announcement was based upon the knowledge that dentine could be cut with less pain in a certain direction than in opposite ones. Unfortunately for the obtaining of correct data, investigations such as Dr. Black's must be made from teeth out of the mouth, and the question, What changes occur from the time the vital connection is severed until the experiment can be performed? is pertinent in the discussion. All the sections of dentine in the investigation were lifeless, and on page 412, under the title, "Pulpless Teeth," are these words: "The majority of pulpless teeth coming into my hands were of fairly bright color, indicating that the pulp was recently dead. These presented no special differences in physical character from those with pulp-tissue intact. But those muddy in color, especially if markedly discolored, showed constantly an additional percentage of water, and the few of which I had the opportunity to test the crushing stress broke below two hundred pounds, showing a marked loss of strength." This was attributed to decomposition of the living matter. What effect the living plasmic material that invades the dentine has upon the strength and density of the tooth has not been proved by the investigations, but clinical observation has long satisfied us, like Dr. Maynard's observation, that the teeth are made more strong and dense and possessed with more resistive qualities by being filled with a material that is in consonance with its physical condition; and when further investigations that Dr. Black refers to are completed there may not be any difference between accepted views as regards the organic matrix and the conclusions of such investigations.

Dr. Black's work cannot be exaggerated for its exactness and magnitude or the value it possesses to the profession; there are many parts to it for study and discussion, and the part I refer to to-night is not exhausted. The whole profession is indebted to him for the undertaking. From a study of it we are stimulated to think more highly of our work as artisans and physicians of the mouth. In the selection of filling-material, however, the choice must still rest upon the basis that clinical experiences have taught, and whether it be of gold, cohesive or non-cohesive, amalgam, tin, gutta-percha, and the various cements, or two in combination, the physical conditions of the patient and of the tooth are as important to consider as skill and ability of manipulation, and the patient is best treated when one is not overshadowed by the significance of the other.

